# Enhancing Mobility: Surgical Deformity Correction and Rehabilitation in Emery-Dreifuss Muscular Dystrophy Type 2

**DOI:** 10.7759/cureus.74805

**Published:** 2024-11-30

**Authors:** Anindya Debnath, Badarinath Athani, Jaya Das, NC Chandini

**Affiliations:** 1 Physical Medicine and Rehabilitation, St. John's National Academy of Health Sciences, Bengaluru, IND

**Keywords:** cpk, edmd type 2, emery-dreifuss muscular dystrophy, genetic diagnosis, heterozygous mutation, lmna gene, omim, post-surgical rehabilitation, surgical rehabilitation, tendo achilles contracture

## Abstract

Emery-Dreifuss Muscular Dystrophy (EDMD) is a rare genetic disorder characterized by muscle weakness, joint contractures, and cardiac dysfunction. Within this spectrum, EDMD Type 2, attributed to a heterozygous missense variant in exon 9 of the LMNA gene, presents a distinctive clinical profile. This case report details the presentation and management of a teenage girl displaying neck, trunk, upper and lower limb weakness, Achilles tendon contracture, and lordosis. Initial assessments, including elevated creatine phosphokinase (CPK) levels and electromyography (EMG) suggestive of myopathy, prompted further investigation. Genomic analysis using targeted gene sequencing using the ExomeDepth method subsequently confirmed the rare autosomal variant of EDMD Type 2 within the OMIM (Online Mendelian Inheritance in Man) phenotype. Given the heterogeneity of EDMD, cardiac evaluation becomes paramount in understanding its multifaceted nature. In this case, the patient underwent surgical correction, specifically, Achilles tendon release, and subsequent post-surgical rehabilitation. Notably, there was a marked improvement in ambulation, underscoring the significance of early release of contractures in enhancing overall functional outcomes. This comprehensive case study not only contributes insights into the clinical, genetic, and surgical aspects of EDMD Type 2 but also highlights the pivotal role of timely interventions in optimizing patient outcomes.

## Introduction

Emery-Dreifuss muscular dystrophy (EDMD), a chronic myopathy first delineated by Emery and Dreifuss in 1966, encompasses a spectrum of genetic disorders with distinct clinical and genetic characteristics [1]. The initial identification led to the recognition of a benign X-linked recessive form, now categorized as EDMD Type 1 (EDMD1). Subsequent insights in the 1980s unveiled another variant of the disorder with autosomal transmission, referred to as EDMD Type 2 (EDMD2). Despite its autosomal dominant inheritance pattern, the prevalence of both forms remains poorly defined, adding a layer of complexity to the understanding of this disorder [1]. Sporadic mutations are rare but are increasingly identified in LMNA. The autosomal dominant and recessive forms of EDMD affect both males and females equally, whereas the X-linked form primarily manifests in males, with some female carriers also exhibiting clinical features [2-4]. Several genes, including EMD, LMNA, SYNE1, SYNE2, FHL1, and TMEM43, have been linked to EDMD and assigned to specific subtypes (EDMD1, EDMD2 and EDMD3, EDMD4, EDMD5, EDMD6, and EDMD7, respectively), as catalogued in the Online Mendelian Inheritance in Man (OMIM) database. Additionally, other genes such as SUN1, SUN2, and TTN have been associated with EDMD. Despite these findings, over 60% of patients do not show mutations in EMD or LMNA, the most commonly implicated genes, suggesting the presence of yet unidentified causative genes [3].

Rooted in mutations within genes coding for nuclear envelope proteins, EDMD manifests through a trio of predominant clinical features-early-onset contractures, progressive muscular atrophy and weakness, and cardiac conduction disturbances. This array of symptoms necessitates a high index of diagnostic suspicion, with clinical and electrocardiographic findings serving as primary indicators. Further confirmation is typically achieved through muscle biopsy and genetic studies, outlining the intricate interplay between clinical presentation and underlying genetic anomalies [2,5].

This introduction sets the contextual backdrop for comprehending EDMD, emphasizing its historical evolution, the diverse genetic basis of its subtypes, and the multifaceted clinical manifestations that characterize this chronic myopathy. The subsequent case report will delve into a specific instance within the EDMD spectrum, shedding light on the complexities of diagnosis, management, and the imperative for a holistic approach in addressing this rare muscular dystrophy. The role of surgical release of Achilles tendon contractures in achieving functional improvement will be a key focus, underscoring its significance in the comprehensive care of individuals with EDMD.

## Case presentation

The 19-year-old female patient, a first-year Pre-University Course (PUC) Arts student hailing from the northern part of India, represents the firstborn in a non-consanguineous union with no similar history in the family. Born through cesarean section following an uneventful antenatal period, she cried at birth with no reported complications such as prolonged labor, asphyxia, meconium-stained liquor, seizures, or jaundice. Breastfed and meeting developmental milestones until the age of six, including gross motor achievements like sitting with support (at five months), crawling (at seven months), standing (at nine months), and walking (at 11 months), she exhibited the ability to walk, run, and climb stairs until the age of six.

When she was six years old, her parents observed increased falls and difficulty ascending stairs, although she retained the ability to descend. Despite being able to sit in a squatting position, rising independently became challenging, necessitating assistance. Issues with toe grip while wearing slippers emerged, and she exhibited self-evacuation for bowel and bladder functions.

Seeking medical attention when she was nine years old, a local clinician recommended a muscle biopsy from the left vastus lateralis muscle. The biopsy revealed adipose tissue infiltration, focal endomysial fibrosis, and myofibers displaying variations in diameter. Internal nuclei, splitting necrosis, macro phagocytosis, and a cluster of regenerating fibers collectively indicated muscular dystrophy. By the age of 10, she was increasingly unable to place her feet flat on the ground while walking and persistently walked on her toes. Her parents noticed that she had more frequent falls and an inability to climb up stairs, although she could climb down. Over the years, she also developed difficulty keeping her knees locked while standing for extended periods. She was able to sit in a squatting position but was unable to get up without help from her parents. She developed a poor toe grip while wearing slippers. She was self-sufficient in evacuating her bowels and bladder. There was a progressive worsening of ankle tightness, and she started walking on the tips of her toes. Her parents consulted multiple doctors without receiving a conclusive diagnosis.

**Figure 1:**
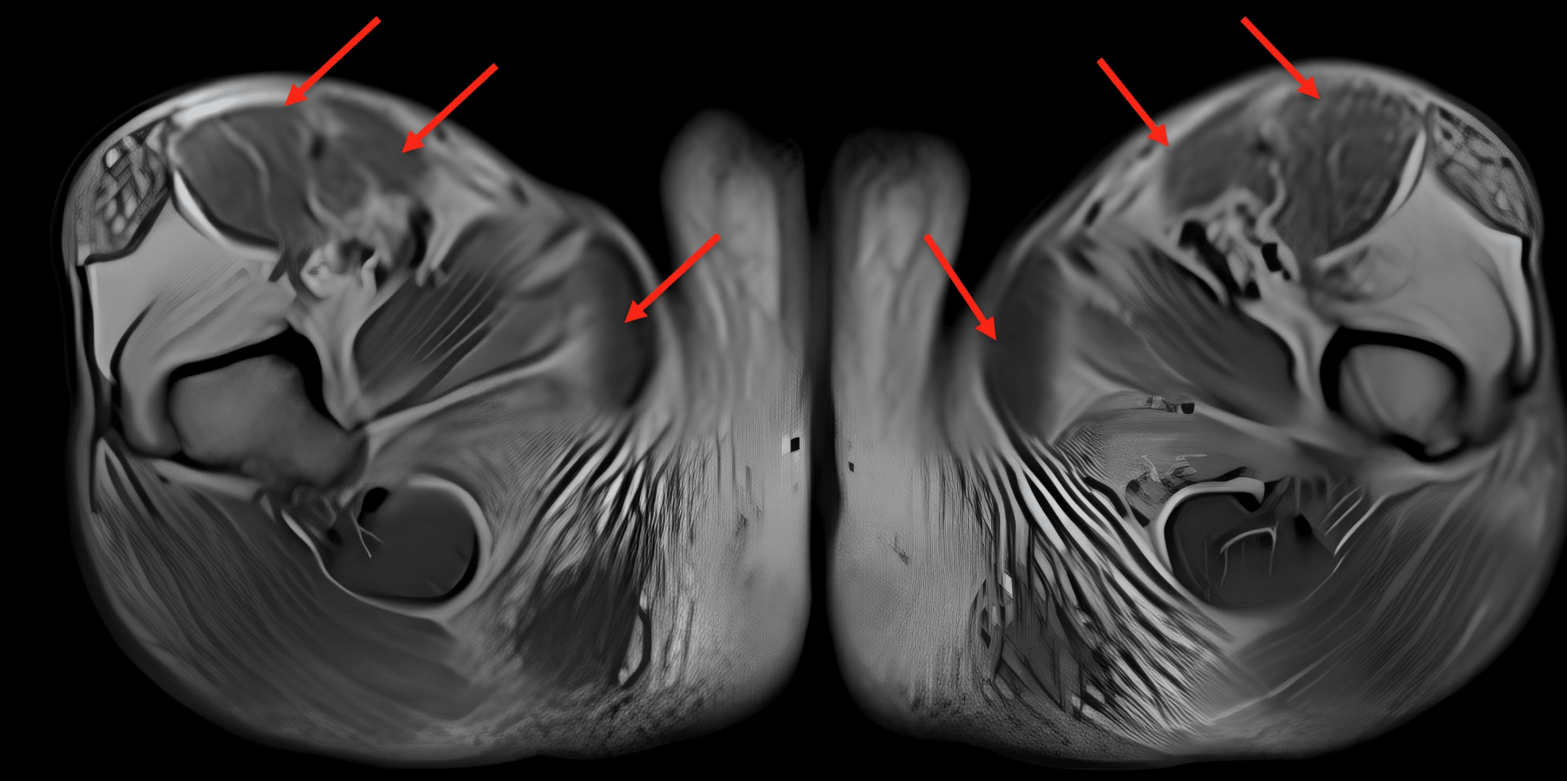
MRI bilateral thigh. MRI showed fatty infiltration in bilateral thighs with relative sparing of bilateral gracilis, sartorius, and rectus femoris muscles (medial to lateral arrows) in each side.

Further assessment at a higher medical center in September 2023 included an MRI of her lower limbs, unveiling moderate fatty infiltration in bilateral thighs with relative sparing of bilateral gracilis, sartorius, and rectus femoris muscles. Severe fatty replacement manifested in bilateral soleus muscles (Figure 1). Referral to the Physical Medicine and Rehabilitation (PMR) department for further management and rehabilitation followed.

Targeted gene sequencing was done using the ExomeDepth method [6]. The sequences obtained are aligned to the human reference genome (GRCh38) using a Burrows-Wheeler aligner (Sentieon, San Jose, CA) [7], uncovering an LMNA gene mutation at Exon 9 (c.1583C>G variant), indicating a heterozygous autosomal dominant type of EDMD2 (Table 1).

**Table 1:** Genetic testing. Variant description: A heterozygous missense variant in exon 9 of the LMNA gene (chr1:g.15613 7207C>G; Depth: 220x) that results in the amino acid substitution of arginine for threonine at codon 528 (p.Thr528Arg; ENSTOO000368300.9) was detected. The observed variant lies in the Sema domain of the LMNA protein. Online Mendelian Inheritance in Man (OMIM) phenotype: Emery-Dreifuss muscular dystrophy 2 (OMIM#181350) is caused by heterozygous mutations in the LMNA gene (OMIM*150330). This disorder is characterized by myopathic changes in certain skeletal muscles and early contractures at the neck, elbows, and Achilles tendons, as well as cardiac conduction defects. Hence, this LMNA variation is classified as a likely pathogenic variant.

Gene (transcript)	Location	Variant	Zygosity	Disease (OMIM)	Inheritance	Classification
LMNA (+); ENST00000368300.9	Exon 9	c.1583C>G	Heterozygous	Emery-Dreifuss muscular dystrophy 2; OMIM#181350	Autosomal dominant	Likely pathogenic

Blood investigations indicated elevated CPK levels (646 U/L) and low plasma vitamin D (11.8 ng/mL) (Table 2).

**Table 2:** Blood workups. Blood investigations showed elevated serum creatine kinase and low vitamin D levels (marked in bold).

Test description	Value	Unit	Biological reference range
Serum creatine kinase	646	U/L	29-168
Serum lactate dehydrogenase	162	U/L	125-220
Serum calcium	9.1	mg/dL	8.4-10.2
Plasma vitamin D	11.8	ng/mL	>30

Cardiac evaluation, including ECG showing sinus tachycardia and an echocardiogram with a normal study and ejection fraction of 65%, revealed additional insights. 

**Figure 2:**
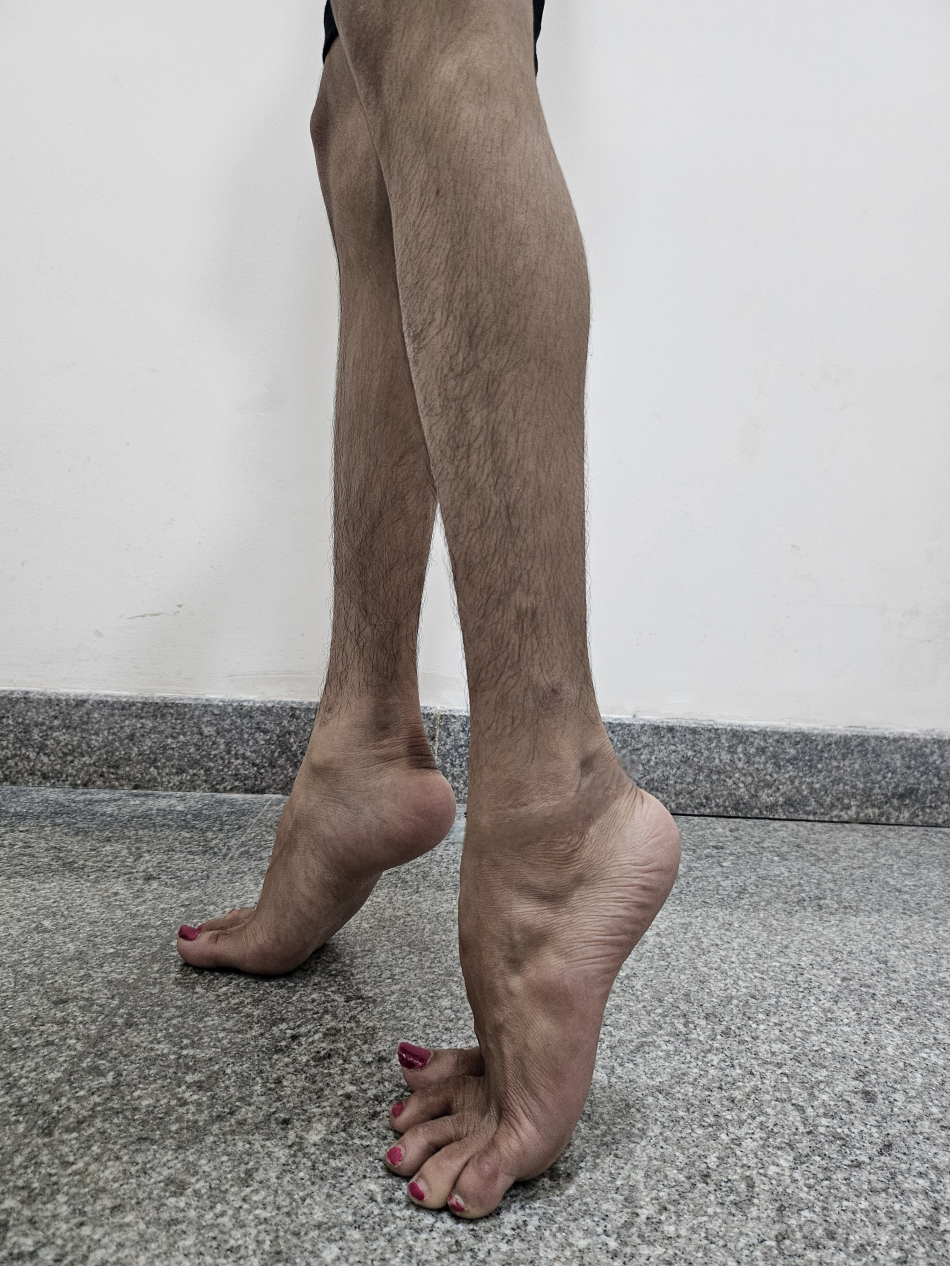
Bilateral ankle contractures on presentation. Fixed equinus deformity of bilateral ankle.

Physical examination highlighted a comprehensive musculoskeletal spectrum, featuring generalized wasting in both upper and lower extremities. Remarkably, certain muscles, including the bilateral deltoid, supraspinatus, infraspinatus, sartorius, and rectus femoris, exhibited peculiar sparing. Weakness extended to both neck and trunk musculature, with symmetric weakness of biceps and triceps muscles (Medical Research Council {MRC} scale: 4/5). Bilateral elbow contractures restricted passive range of motion (ROM) of 20-150 degrees, and gastrosoleus contractures impacted ankles, limiting passive ankle ROM of 40-50 degrees of plantar flexion (Figure 2). Depressed deep tendon reflexes in both upper and lower limbs were noted, while sensory examination yielded unremarkable findings. The significant presence of the Gower sign and adoption of a lordotic posture during walking emphasized proximal muscle weakness, contributing to an equinus gait. X-rays were done which showed scoliosis. 

**Figure 3:**
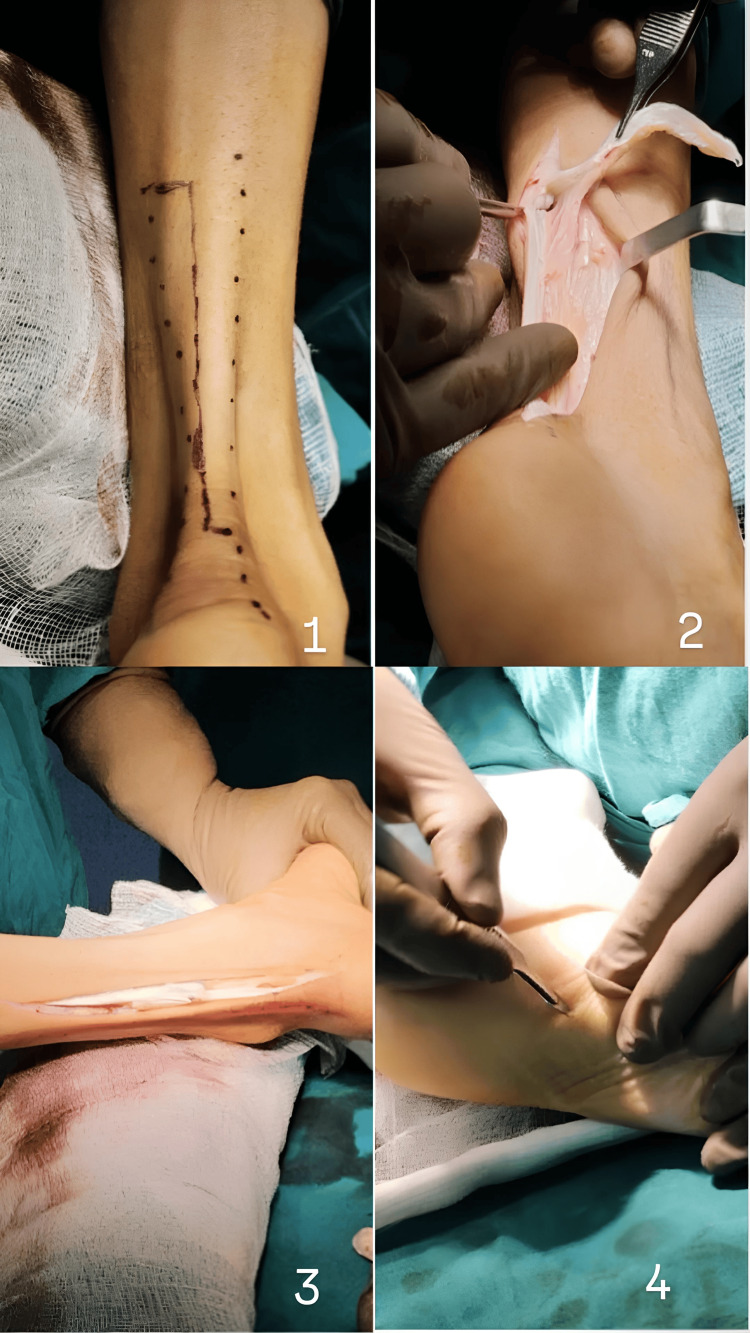
Bilateral Z lengthening of the Achilles tendon and percutaneous plantar fascia release. (1) Surface marking for Z lengthening of the Achilles tendon; (2) Achilles tendon Z lengthening; (3) adequate correction of equinus deformity; and (4) percutaneous plantar fascia release.

The patient’s primary goal was to achieve plantigrade walking and pursue higher education, necessitating significant improvements in mobility and functional independence. Upon admission, the patient’s Functional Independence Measure (FIM) score was 108/126, and her North Star Ambulatory Assessment (NSAA) score was 16/34, highlighting substantial functional limitations, particularly in ambulatory activities. The comprehensive treatment plan was designed to address these limitations and align with the patient's aspirations for enhanced mobility and academic pursuits. The preoperative phase involved thorough preparation to optimize the patient’s physical condition for surgery, including detailed gait assessment, range of motion activities, endurance exercises, and sub-maximal strengthening. The patient underwent a detailed pre-anesthetic assessment and was advised against the use of succinylcholine due to potential complications associated with muscular dystrophies. The surgical intervention included bilateral Z lengthening of the Achilles tendon and percutaneous plantar fascia release (Figure 3), aimed at alleviating contractures and improving flexibility.

**Figure 4:**
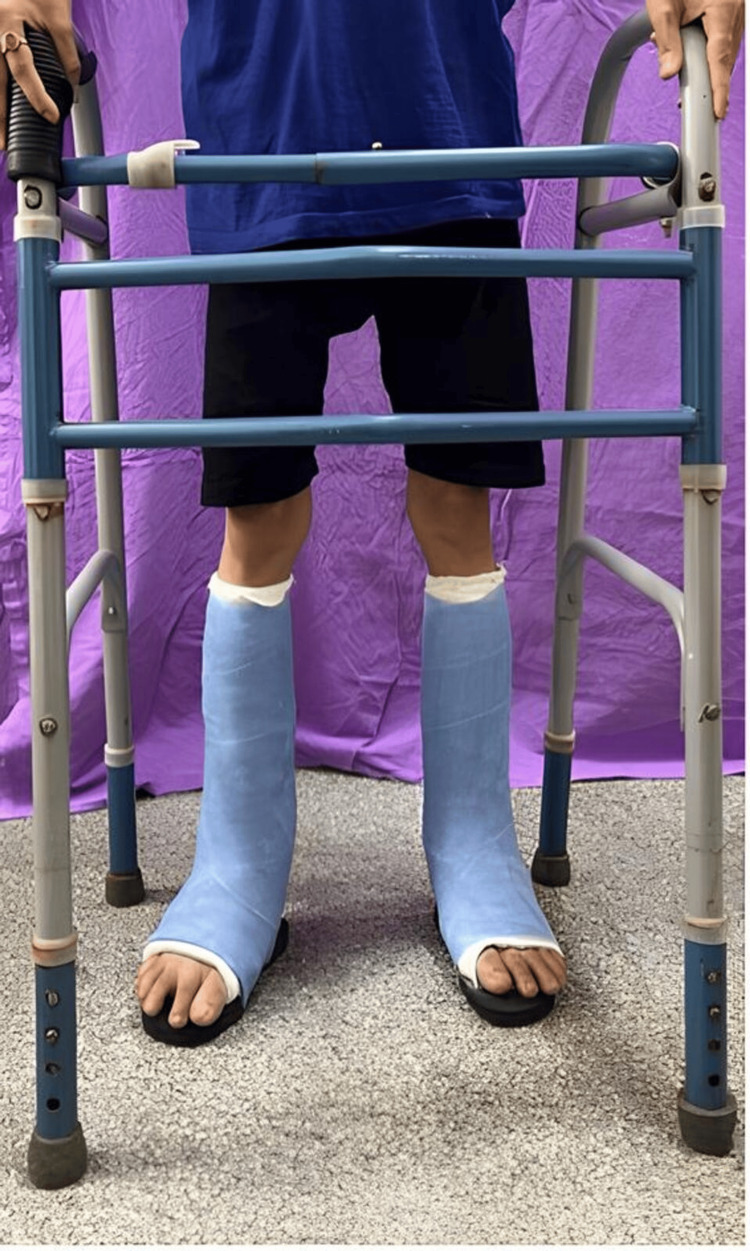
Postoperative rehabilitation. Post-surgical walking cast and gait training with walker support.

Immediate postoperative care involved below-knee casting to stabilize the surgical corrections, with early mobilization initiated 48 hours post-surgery using a walker to promote circulation and prevent complications. Bilateral ankle-foot orthoses (AFOs) supported the ankles and facilitated proper gait mechanics during recovery. Structured gait training sessions focused on improving walking ability, initially with a walker and then progressing to elbow crutches (Figure 4).

**Figure 5:**
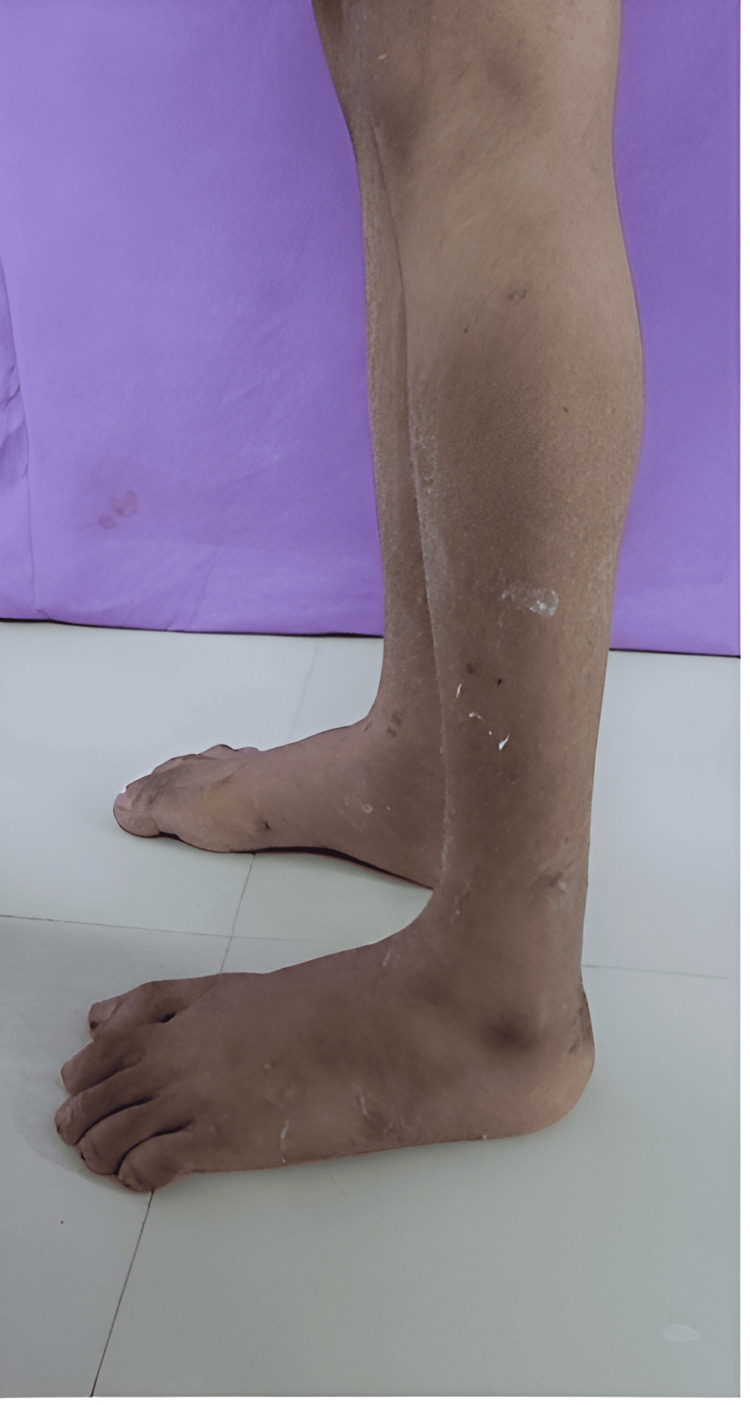
Postoperative correction of equinus deformity. Achieving plantigrade foot post-surgical correction.

The patient was discharged after demonstrating significant improvements in walking ability, ultimately achieving plantigrade foot (Figure 5) and independent ambulation, a crucial milestone aligning with her personal goals. On discharge, her FIM score was 113/126 and her NSAA score was 22/34. 

The comprehensive approach, integrating preoperative rehabilitation, precise surgical intervention, and diligent postoperative care, led to significant improvements in the patient’s ambulatory capabilities. The early release of contractures was crucial in enhancing functional outcomes, highlighting the importance of timely and targeted interventions in EDMD2 management. This case underscores the potential for substantial quality-of-life improvements in EDMD2 patients through a coordinated, multidisciplinary approach. Emphasizing the need for individualized treatment plans, this approach addresses both the physical and aspirational needs of patients, ultimately enabling them to achieve their personal and functional goals.

## Discussion

A high index of suspicion for EDMD is crucial given its presentations, as timely and accurate diagnosis can significantly influence management and outcomes. Managing contractures in Emery-Dreifuss muscular dystrophy (EDMD) presents unique challenges, with limited detailed surgical descriptions available in existing literature, especially for lower limbs. Previous publications have predominantly addressed elbow contractures in EDMD, which, although typically not exceeding 35 degrees, can severely impact function when more pronounced. Surgical interventions for elbow contractures, including anterior release and selective muscle lengthening, have been reported with positive outcomes. However, Achilles tendon contractures in EDMD remain underreported. This highlights the importance of recognizing and addressing less common contractures in EDMD to achieve optimal functional outcomes [8]. This aligns with our findings and reinforces the value of surgical intervention in managing EDMD-related contractures. During surgery, we encountered extensive fibrosis of the gastrosoleus muscle, indicating its non-functionality. By lengthening the functional muscles and transecting the non-functional Achilles tendon, we successfully improved the functional range of motion without compromising perceived strength. This meticulous approach resulted in a significant improvement in the patient’s ankle range of motion, from 50 degrees of plantar flexion to 20 degrees of dorsiflexion. Postoperative assessments revealed improved gait, and the patient reported considerable functional improvement without ongoing hindrance from her contractures. At the six-month follow-up, the patient demonstrated significant functional improvement, actively performing all daily activities independently. She successfully resumed her higher education and expressed immense satisfaction with the intervention, which helped her achieve her felt needs and pursue her academic goals. 

Addressing the systemic complications that EDMD poses requires good clinical knowledge. The patient and her family were counseled about family screening, the possible recurrence of contractures, and cardiac complications as she ages. Regular follow-ups, including yearly ECG and echocardiogram, were recommended to monitor her condition and manage any arising issues promptly.

## Conclusions

This case underscores the importance of a coordinated, multidisciplinary approach to managing EDMD. Our findings highlight the potential for substantial quality-of-life improvements in EDMD patients through individualized treatment plans that address both physical and aspirational needs. With accurate diagnosis and appropriate cardiac precautions, patients with EDMD can typically survive into middle age or beyond. Contracture release, when deemed necessary, can significantly enhance their function and independence, as evidenced by our patient’s progress. This case adds to the limited body of literature on surgical management in EDMD, emphasizing the need for detailed surgical descriptions and highlighting the significant impact of such interventions on patient outcomes.
